# Analysis of influencing factors of multiple urethrocutaneous fistula after urethroplasty in children with hypospadias

**DOI:** 10.3389/fped.2023.1103200

**Published:** 2023-03-21

**Authors:** Guangchao Tian, Bingtao Guo, Lihua Zhang

**Affiliations:** Department of Pediatric Urology, The Third Affiliated Hospital of Zhengzhou University, Zhengzhou, China

**Keywords:** hypospadias/SU, hypospadias/CO, urethrocutaneous fistula, multiple fistula, complications, risk factors

## Abstract

**Objective:**

The objective of this study was to investigate the influencing factors of multiple urethrocutaneous fistula (UF) after urethroplasty in children with hypospadias.

**Methods:**

The clinical data of 195 children with UF after urethroplasty treated surgically in the Third Affiliated Hospital of Zhengzhou University from August 2015 to August 2022 were retrospectively analyzed and divided into the single UF group (*n* = 134) and the multiple UF group (*n* = 61) according to whether multiple UF occurred after urethroplasty. The possible correlated factors were collected and compared between the two groups, including hypospadias degree, length of formed urethra, time of urethroplasty, pre-urethroplasty weight, age at urethroplasty, urethroplasty style, season of urethroplasty, the first fistula repair method, season of the first fistula repair, diameter of the largest fistula of the first fistula repair, time of the first fistula repair surgery, and other 13 factors.

**Results:**

By univariate analysis, statistically significant differences were found between the two groups in age at urethroplasty, length of the formed urethra, method of urinary drainage after urethroplasty, whether or not purulent urethral drainage after first fistula repair was present, the first fistula repair method, and diameter of the largest fistula of the first fistula repair (*P* < 0.05). After multifactorial analysis, the independent risk factors associated with multiple UF after urethroplasty were determined to be use of a vesicostomy tube as the urinary drainage method after urethroplasty (*P* < 0.05, OR = 6.574, 95% CI: 2.720–15.891) and the presence of purulent urethral drainage after first fistula repair (*P* < 0.05, OR = 2.723, 95% CI: 1.214–6.109).

**Conclusions:**

A catheter as the drainage method after urethroplasty is an independent protective factor for multiple urethrocutaneous fistula, and the existence of purulent urethral secretions after the first fistula repair is an independent risk factor.

## Introduction

Hypospadias is the most common congenital malformation of male external genitalia, and its incidence is significantly different in different regions worldwide (1–4). The cause of hypospadias is not clear, and most scholars believe that it is the result of the comprehensive influence of multiple factors, so it is impossible to achieve targeted prevention. In addition, the diagnosis and surgical treatment of fetal hypospadias have obvious limitations, so postnatal surgery is the only solution for it (5, 6). With the development of pediatric anesthesia and surgical instruments in recent years, the operation of hypospadias in children has become mature. However, the postoperative complications cannot be completely avoided. Urethrocutaneous fistula (UF) is one of the common short-term complications after urethroplasty in children with hypospadias. The incidence of UF has decreased to some extent in recent years, but it is still much higher than other complications. The high incidence of UF and the high possibility of recurrence after the first fistula repair are still key problems to be solved in pediatric urology. The purpose of this study was to explore the influencing factors of multiple UF after urethroplasty in children with hypospadias.

## Methods

### Study population

We conducted a retrospective case–control study using our hospital's clinical data on patients with UF after urethroplasty from August 2015 to August 2022, which was approved by the Ethics Committee of the Third Affiliated Hospital of Zhengzhou University. The exclusion criteria were as follows: (1) complicated with DSD (disorder of sexual development); (2) initial surgery such as penile straightening or urethroplasty was performed in other hospital; (3) initial surgery was performed in our hospital, and complications such as UF were treated in other hospital; (4) history of application of sex hormones such as chorionic gonadotropin and testosterone undecanoate within 3 months before urethroplasty (7); (5) complicated with urethral stricture when UF occurred for the first time; (6) the fistula was located in the thin ventral tissue of distal part of the glans, and the connecting tissue between the fistula and the external orifice of the urethra was cut directly, without glansplasty; (7) incomplete data.

### Data collection

The clinical data of 195 children with UF after urethroplasty meeting the inclusion criteria were collected. These cases were divided into the single UF group (*n* = 134) and the multiple UF group (*n* = 61) according to whether multiple UF occurred after urethroplasty (Table 1). Twenty-four factors related to multiple UF were collected: degree of hypospadias (I, II, III, IV) (8), urinary comorbidities (cryptorchidism, hydrocele concurrenting, etc.), length of formed urethra, time of urethroplasty, pre-urethroplasty weight, age of urethroplasty, urethroplasty style [staged operation (one-stage penile straightening and two-stage Snodgrass), Snodgrass, Duckett and Duplay, Snodgrass and Ducket, Koyanagi, Duckett], season of urethroplasty (spring, summer, autumn, winter), the presence of purulent urethral drainage after urethroplasty, existing usage of analgesics or sedatives after urethroplasty, method of urinary drainage after urethroplasty, time of removal of catheter after urethroplasty, time of removal of penile dressing after urethroplasty, the first fistula repair method, season of the first fistula repair, diameter of the largest fistula of the first fistula repair, time of the first fistula repair surgery, weight of the first fistula repair surgery, interval time between urethroplasty and the first fistula repair surgery, number of UF of the first fistula repair, the presence of purulent urethral drainage after first fistula repair, existing usage of analgesics or sedatives after the first fistula repair, time of removal of catheter after the first fistula repair, and time of removal of penile dressing after the first fistula repair.

**Table 1:** Demographic characteristics of the single UF group and multiple UF group.

	Single UF group (*n* = 134)	Multiple UF group (*n* = 61)	*Z*	*P-*value
Pre-urethroplasty weight [*M* (*Q_1_*, *Q_3_*), kg]	15 (12, 20)	16 (14, 21)	−1.418	0.156
time at urethroplasty [*M* (*Q_1_*, *Q_3_*), min]	102 (85, 124)	100 (77.5, 117.5)	−1.072	0.284
Time of removing penile dressing after urethroplasty [*M* (*Q_1_*, *Q_3_*),d]	6 (6, 7)	6 (6, 6)	−1.476	0.14
Time of removing catheter after urethroplasty [*M* (*Q_1_*, *Q_3_*),d]	14 (14, 15)	14 (14, 14)	−1.182	0.237
Number of UF of the first fistula repair [*M* (*Q_1_*, *Q_3_*)]	1 (1, 2)	1 (1, 2)	−0.56	0.576
Interval time between urethroplasty and the first fistula repair surgery [*M* (*Q_1_*, *Q_3_*), months]	12 (11, 16)	12 (10.5, 12.5)	−1.253	0.21
Weight of the first fistula repair surgery [*M* (*Q_1_*, *Q_3_*), kg]	19 (14, 24.125)	18 (15.6, 24)	−0.629	0.53
Time of the first fistula repair surgery [*M* (*Q_1_*, *Q_3_*), min]	43 (32.75, 56.25)	48 (30.5, 69)	−1.414	0.157
Time of removing penile dressing after the first fistula repair [*M* (*Q_1_*, *Q_3_*), days]	6 (5, 6)	6 (6, 6)	−1.657	0.098
Time of removing catheter after the first fistula repair [*M* (*Q_1_*, *Q_3_*), days]	14 (12, 14)	14 (14, 14)	−1.262	0.207
age at urethroplasty [*M* (*Q_1_*, *Q_3_*), month]	36 (24, 67.25)	48 (29, 77.5)	−2.028	0.043
Length of formed urethra [*M* (*Q_1_*, *Q_3_*), cm]	3 (2.5, 4)	3.5 (3, 4)	−2.104	0.035

UF, urethrocutaneous fistula.

### Statistical analysis

Multivariate logistic regression analysis was used to analyze the influencing factors significantly correlated with multiple UF after urethroplasty in children with hypospadias. The Shapiro–Wilk test was used to test the normality of quantitative data. Continuous variables were represented as medians and interquartile ranges [*M* (*Q*_1_, *Q*_3_)] if non-normally distributed. The 12 continuous variables in this study were non-normal distribution and analyzed by the Wilcoxon rank-sum test on both sides. Qualitative data were analyzed by the *χ*^2^-test and represented as frequencies and percentages [*n* (%)] on both groups. Statistical analyses were performed using SPSS v.25 software. Statistical significance was set at *P* < 0.05.

## Results

None of the 195 children with UF were complicated with urethral diverticulum in this study. A total of 134 children with single UF were cured after one time of fistula repair. Among the 61 children with multiple UF, 39 cases were cured after two times of fistula repair, 21 cases were cured after three times of fistula repair, and 1 case was cured after four times of fistula repair.

By univariate regression analysis, statistically significant differences were found between the single UF group and the multiple UF group in age at urethroplasty, length of the formed urethra, method of urinary drainage after urethroplasty, the presence of purulent urethral drainage after first fistula repair, the first fistula repair method, and the diameter of the largest fistula of the first fistula repair (*P* < 0.05) (Table 2).

**Table 2:** Univariate regression analysis of multiple UF after urethroplasty.

		Single UF group (*n* = 134)	Multiple UF group (*n* = 61)	Z/*χ*^2^	*P*-value
The presence of purulent urethral drainage after first fistula repair [*n* (%)]	Yes	96 (71.6)	33 (54.1)	5.762	0.016
	No	38 (28.4)	28 (45.9)		
Method of urinary drainage after urethroplasty [*n* (%)]	Catheter	63 (47.0)	12 (19.7)	13.241	<0.001
	Vesicostomy tube	71 (53.0)	49 (80.3)		
The first fistula repair method [*n* (%)]	Ligation suture	70 (52.2)	11 (18.0)	23.168	0.001
	direct contraposition suture	29 (21.7)	25 (41.0)		
	Snodgrass	22 (16.4)	14 (23.0)		
	Ligation suture and direct contraposition suture	4 (3.0)	2 (3.4)		
	Ligation suture and Snodgrass	7 (5.2)	5 (8.3)		
	Direct contraposition suture and Snodgrass	2 (1.5)	3 (4.9)		
	Ligation suture and direct contraposition suture and Snodgrass	0 (0.0)	1 (1.7)		
Diameter of the largest fistula of the first fistula repair [*n* (%)]	<3 mm	78 (58.2)	13 (21.3)	23.073	<0.001
	≥3 mm and <15 mm	38 (28.4)	31 (50.8)		
	≥15 mm	18 (13.4)	17 (27.9)		
Age at urethroplasty [*M* (*Q_1_*, *Q_3_*), months]	36 (24, 67.25)	48 (29, 77.5)	−2.028	0.043	
Length of formed urethra [*M* (*Q_1_*, *Q_3_*), cm]	3 (2.5, 4)	3.5 (3, 4)	−2.104	0.035	

UF, urethrocutaneous fistula.

The statistically significant factors calculated by the univariate regression analysis were added in the multivariate logistic regression analysis. The results showed that the factors related to multiple UF after urethroplasty were method of urinary drainage after urethroplasty and the presence of purulent urethral drainage after first fistula repair (*P* < 0.05)(Table 3).

**Table 3:** Multivariate logistic regression analysis of multiple UF after urethroplasty.

		B	SE	Wald	df	*P-*value	OR (95% CI)
The presence of purulent urethral drainage after first fistula repair [*n* (%)]	Yes	1.002	0.412	5.904	1	0.015	2.723 (1.214–6.109)
	No	0.000					1.000
Method of urinary drainage after urethroplasty	Vesicostomy tube	1.883	0.450	17.487	1	<0.001	6.574 (2.720–15.891)
	Catheter	0.000					1.000
The first fistula repair method			3.473	6	0.748	
Ligation suture	0.000					1.000
Direct contraposition suture	0.982	0.885	1.234	1	0.267	2.671 (0.472–15.122)
Snodgrass	0.131	0.995	0.017	1	0.895	1.140 (0.162–8.011)
Ligation suture and direct contraposition suture	0.165	1.246	0.018	1	0.894	1.180 (0.103–13.559)
Ligation suture and Snodgrass	0.272	1.056	0.066	1	0.797	1.312 (0.166–10.407)
Direct contraposition suture and Snodgrass	1.384	1.404	0.972	1	0.324	3.990 (0.255–62.516)
Ligation suture, direct contraposition suture, and Snodgrass	23.763	40,192.969	0.000	1	1.000	20,896,647,559.040
Diameter of the largest fistula of the first fistula repair			3.007	2	0.222	
<3 mm	0.000					1.000
≥3 mm and <15 mm	1.083	0.847	1.637	1	0.201	2.955 (0.562–15.539)
≥15 mm	1.701	0.983	2.993	1	0.084	5.482 (0.798–37.675)
Age at urethroplasty	0.004	0.005	0.600	1	0.439	1.004 (0.994–1.014)
Length of formed urethra	0.131	0.165	0.625	1	0.429	1.140 (0.824–1.576)

UF, urethrocutaneous fistula.

## Discussion

UF is one of the most common complications after urethroplasty in children with hypospadias. At present, there are many studies on the incidence of the first UF, but there are few on the incidence of recurrent UF after the first fistula repair. The multiple UF incidence reported by Abdullaev et al., Yassin et al., and Aldaqadossi et al. are 19%, 22.3%, and 4.4%, respectively (9–11). However, the sample size in their studies on multiple UF was small, including 63 cases in Abdullaev et al. and 67 cases in Yassin et al., and only 45 cases in Aldaqadossi et al., all of which are retrospective studies, so the conclusions are not completely reliable. In our study, 195 children with UF who met the criteria were enrolled, of which 134 cases had no recurrence after the first fistula repair and 61 cases had recurrence after the first fistula repair. The incidence of multiple UF in our study was slightly higher than others, which may be related to the differences in the sample inclusion criteria, surgeon's surgical techniques, surgical methods, and sample size.

The formation of UF after urethroplasty is related to many factors, such as the surgical experience of surgeons, urethroplasty style, method of urinary drainage after urethroplasty, length of the formed urethra, infection at the operation site, dysvascularization of formed urethral tissue, necrosis and inactivation of skin flap, and hypoplasia of glans (12, 13). There are many studies on the influencing factors of the initial UF but few on the multiple UF. Hypospadias degree, length of formed urethra, and the type of material used to cover the fistula are determined to be related to the recurrence of UF (9, 11). Our study suggests the vesicostomy tube as a method of urinary drainage after urethroplasty and the existence of purulent urethral secretions after the first fistula repair will lead to multiple UF. It is controversial to use a catheter or a vesicostomy tube to drain urine after urethroplasty or the fistula repair surgery currently (14). The results of our study support the conclusion that catheter is the better way for urine drainage, and the accumulation of surgical experience of surgeons is also of certain significance for the prevention of UF occurrence after urethroplasty (15). However, our study does not take into account the accumulation of surgical experience of surgeons, which makes the conclusions of this study not rigorous to a certain extent. This study is a retrospective case–control study, which cannot completely eliminate the influence of confounding factors such as the accumulation of surgical experience of surgeons, so it needs to be verified by a prospective follow-up research study regarding multiple UF. Purulent urethral secretion is caused by bacterial infection in the tissue of operation site after urethroplasty or fistula repair surgery, which will cause necrosis of the skin flap tissue cell in the microenvironment of bacteria, pus, and inflammatory factors, and then cause the first and multiple UFs.

The local application of the growth factor and prolonging the indwelling time of a catheter is sometimes used to treat acicular UF in clinics. Surgery is still the main method to treat acicular UF because of the lack of reliable research support. Srivastava et al. divided UF into two types according to its diameter. UF with a diameter smaller than 2 mm was repaired by direct contraposition suture, and that bigger than 2 mm was repaired by transferring a skin flap from other sites (16). In the traditional method, the superficial skin tissue of the penis around the large UF is dissociated, and then a local propulsive flap is formed to suture the fistula. In the traditional method, the blood supply of the penile skin tissue around the UF is always poor because the microvessels present there are likely to have been destroyed by electrocoagulation during urethroplasty or first fistula repair surgery (17). We evaluated the diameter, location, number of UF, and blood supply of the penis skin tissue around the UF, and then adopted a comprehensive method to repair the first or multiple UF. The key step of our comprehensive method to prevent recurrence of UF was to cover the repaired UF using the tissue with good blood supply (such as testicular sheath and dartos fascia) (18). The tissue conditions around the UF of children who have experienced fistula repair surgery multiple times are always very poor, which is also an important reason why children with UF will still recur after repeated fistula repair (8). Carmignani used microscope magnification technology to achieve the tight suture of UF and protect the microvessels, so as to reduce the recurrence of UF (19). For children with multiple UF whose local blood supply around UF is poor, and the adjacent tissue that can be used for skin flap to repair UF is nearly exhausted, some scholars use biosynthetic materials (such as Integra®, bladder mucosal acellular matrix, and intestinal mucosal acellular matrix) to repair (20–22). Both the microscope magnification technology and the biosynthetic materials can be used as an alternative method to repair the multiple UF whose local blood supply and adjacent tissue that can be used for the skin flap is poor.

## Conclusion

Our study shows that a catheter used as the drainage method after urethroplasty is an independent protective factor for multiple UF, and the existence of purulent urethral secretions after the first fistula repair is an independent risk factor. In addition, multiple factors such as UF diameter, number, local blood supply around UF, and adjacent tissue conditions should be comprehensively considered when repairing multiple UF.

## Data Availability

The raw data supporting the conclusions of this article will be made available by the authors, without undue reservation.
